# Repellent, Lethal Activity, and Synergism of *Cannabis sativa* Extracts with Terpenes Against a Laboratory Colony of *Triatoma infestans*

**DOI:** 10.3390/plants14213258

**Published:** 2025-10-24

**Authors:** Martín M. Dadé, Martín R. Daniele, Sergio Rodriguez, Pilar Díaz, Maria Pía Silvestrini, Guillermo R. Schinella, Gustavo H. Marin, Daniel Barrio, Jose M. Prieto Garcia

**Affiliations:** 1 Instituto de Ciencias de la Salud, UNAJ-CICPBA, Florencio Varela 1888, Argentina; schinell@med.unlp.edu.ar; 2 Escuela de Veterinaria y Producción Agroindustrial, Universidad Nacional de Río Negro, Choele Choel 8360, Argentina; mrdaniele@unrn.edu.ar; 3 Facultad de Ciencias Médicas, Universidad Nacional de la Plata, La Plata 1900, Argentina; 4 Carrera de Veterinaria, Universidad de Ciencias Empresariales y Sociales (UCES), Cañuelas 1814, Argentina; 5 Asociación Civil Cultivar Ciencia Argentina, La Plata 1900, Argentina; sgrodriguez01@gmail.com (S.R.); pilidiazprat28@gmail.com (P.D.); 6 Facultad de Ciencias de la Salud, Instituto de Investigación de Ciencias de la Salud, Universidad del Centro de la Provincia de Buenos Aires, Olavarría 7400, Argentina; pia.silvestrini@salud.unicen.edu.ar; 7 CIT Río Negro, Universidad Nacional de Río Negro, Viedma 8500, Argentina; drbarrio@unrn.edu.ar; 8 Centre for Natural Products Discovery, School of Pharmacy and Biomolecular Sciences, Liverpool John Moores University, Liverpool L3 3AF, UK

**Keywords:** *Cannabis sativa*, *Triatoma infestans*, bio-insecticides, cannabinoids, terpenes, vector control

## Abstract

*Triatoma infestans* is one of the primary vectors of Chagas disease. This vector has developed increasing resistance to pyrethroids, the main insecticides used for its control. Recent studies have highlighted the repellent and lethal effects of *Cannabis sativa* on insects, suggesting its potential use in pest management. Based on this, we hypothesize that *C. sativa* could be a viable bioactive for controlling *T. infestans*. To test this hypothesis, acetone and ethanol extracts were obtained from the inflorescences of *C. sativa* L. (Deep Mandarine variety) using sonication. These extracts were analyzed through gas chromatography and high-performance liquid chromatography. The repellent and lethal effects of the extracts were evaluated on fifth-instar nymphs of *T. infestans* from a laboratory colony, as well as on the beneficial non-target species, *Apis mellifera*. The most abundant terpenes identified were *β*-caryophyllene and *β*-pinene, with concentrations exceeding 100 ppm in both extracts. Cannabidiol and Δ^9^-tetrahydrocannabinol were the predominant cannabinoids. Both extracts exhibited maximum lethal activity 48 h after insect contact, with the acetone extract demonstrating a potency five times greater than the ethanolic extract. Binary combinations of *C. sativa* extracts with major terpenes showed dose-dependent interactions against *T. infestans*, ranging from strong synergy (e.g., AE + *β*-caryophyllene, CI = 0.06–0.17) to marked antagonism (e.g., AE + *E*-ocimene, CI = 1.60–4.80). Furthermore, the acetone extract showed a more effective repellent action compared to the ethanol extract, even outperforming N,N-Diethyl-meta-toluamide (DEET, positive control). At a concentration of 25 µg/cm^2^ for 60 min, the acetone extract achieved a 100% repellent effect, whereas DEET required a concentration of 50 µg/cm^2^ to achieve the same effect. Unlike imidacloprid (positive control), neither extract showed toxicity to adult *A. mellifera* at the evaluated doses.

## 1. Introduction

Chagas disease is an infectious disease caused by the protozoan *Trypanosoma cruzi* (Kinetoplastida: Trypanosomatidae). The parasite is transmitted to over 100 mammalian species, including humans, via triatomine insects. While Chagas disease is endemic to 21 countries in the Americas, increasing population mobility has led to its detection in 44 countries [1]. Currently, an estimated 6 to 7 million people are infected with *T. cruzi*, with 75 million at risk of contracting the parasite: approximately 12,000 deaths reported annually. The disease progresses through two phases: acute and chronic. In the chronic phase, approximately one-third of infected individuals may develop the characteristic pathologies of the disease, including cardiac, digestive, and neurological disorders [1].

Chagas disease is considered a neglected disease, mainly due to the lack of interest from private industry in the development of vaccines, antiparasitic, insecticides, and methods of diagnosis of the disease. Given this situation, the WHO established that the main strategy to avoid the presence of newly infected people is the elimination of insect vectors [2]. Pyrethroid insecticides have been used for over 50 years for the chemical control of triatomine insects. Over the past 30 years, the presence of pyrethroid-resistant *T. infestans* populations has been documented in Argentina, Bolivia, Brazil, and Peru [3,4]. This growing resistance highlights the urgent need for new molecules with lethal and sub-lethal activity against *T. infestans*, enabling their rotation and/or combination with currently used insecticides. Previous research has primarily focused on synthetic insecticides, including neonicotinoids, organophosphates, macrocyclic lactones, and formamidines [5,6,7,8]. However, due to their high specificity, safety, biodegradability, and cost-effectiveness, there has been an increasing focus on exploring plant-derived compounds for insect control.

In triatomine insects, the activity of plant-derived molecules has been studied in the form of extracts, essential oils, or individual active compounds. For instance, hexane extracts from leaves and fruits of *Schinus molle* have demonstrated high repellent activity against *T. infestans* nymphs [9], as have acetone extracts from the fruits of *Melia azedarach* [10]. Regarding essential oils, Mojica et al. reported repellent activity against fifth-instar nymphs of *T. infestans* using essential oil from the leaves of *Laurelia sempervirens* (Chilean laurel) [11]. Additionally, monoterpenes such as linalool and menthol have been shown to induce hyperactivation in *T. infestans* nymphs [12].

*Cannabis sativa* L. is an annual dioecious plant from the Cannabaceae family [13], known for its highly diverse chemical composition. The plant’s secondary metabolites include the unique class of phytocannabinoids, accompanied by stilbenoids, flavonoids, terpenoids, alkaloids, lignanamides, and phenolic amides [14,15]. It is estimated that the use of *C. sativa* began over 4000 years ago, initially for producing ropes and textiles. Over time, its applications extended to medicine, religious rituals, and recreational use [16]. Modern medicinal applications of *C. sativa* include the treatment of refractory epilepsy [17], while its anti-inflammatory, analgesic, anxiolytic, and antiemetic properties have been extensively documented and widely utilized, particularly in oncology patients [18]. In recent years, growing evidence of the deterrent effects of *C. sativa* on insects has underscored its potential in pest control [19,20,21].

The composition of *C. sativa* has been the subject of intense research over the last 30 years. Significant strides have been made with the advent of metabolomic tools, such as ultra-performance liquid chromatography and MSn detection [22], leading to the discovery of numerous secondary metabolites [23]. This progress has even led to the coining of the term “Cannabinomics” [24], which is now applied to the chemotaxonomy of its several hundred known varieties, a field referred to as “Authentomics” [25].

Yet, it is hoped that integrating these approaches with cheminformatics tools for compound identification and mass spectral libraries would facilitate the discovery of numerous new phytocannabinoids [22]. To complicate matters further, the postharvest operations of Cannabis materials lead to changes in its composition that can nowadays be better understood with advanced analytical techniques [26].

Considering the pressing need for eco-friendly products to manage disease vectors, *C. sativa* represents a promising avenue of research. In this study, our objectives are to evaluate the lethal and repellent activity of chemically well-defined *C. sativa* extracts against fifth-instar nymphs of *T. infestans* and to assess their selective toxicity towards beneficial non-target insect species.

## 2. Materials and Methods

### 2.1. Chemicals

Technical grade insecticides (imidacloprid and deltamethrin), the repellent (N,N-Diethyl-meta-toluamide). and analytical-grade solvents (acetone and ethanol) were purchased from Sigma-Aldrich (St. Louis, MO, USA). Certified cannabinoid and terpenes reference standard were purchased from Cerilliant (Round Rock, TX, USA) and Restek Corporation (Bellefonte, PA, USA), respectively.

### 2.2. Biological Material

#### 2.2.1. Plant Material

The inflorescences used in the investigation were provided by PlantAR Ciencia (License 46875). The plant material used corresponds to female inflorescences of the *Cannabis sativa* L., Deep Mandarine strain, from the “Delicious Seeds” seed bank (TH Passion, Valencia, Spain). Briefly, the plants were multiplied by cloning from cuttings of mother plants and developed, both in the vegetative and flowering stages, in indoor cultivation with controlled variables (temperature, light, air, and humidity). The growth and development in the vegetative state took eighty days and the flowering stage sixty days. Plant material was stored in vacuum-sealed embossed bags.

#### 2.2.2. Ultrasound Assisted Extraction (UAE)

To obtain the acetone and ethanol extracts, 70 g of freshly ground dry inflorescences were added to 1 L of cold solvent (5 °C) and sonicated in an Q55-110 sonicator (Qsonica, CT, USA) containing ice water at 50% power, 15 s pulses, and 10 min total. The mixture was centrifuged at 5000× *g* for 10 min and the supernatant recovered. The extraction yield was determined as follows: (Dry weight of the solvent extract/dry weight of plant) × 100.

#### 2.2.3. Insects

A colony of *Triatoma infestans* nymphs provided by the Vector and Environment Operational Unit (UnOVE-CeNDIE ANLIS, Buenos Aires, Argentina) was established in 2010 at the Laboratorio de Artrópodos y Vectores (LabArVec, La Plata, Argentina) and has been maintained without exposure to any insecticides since.

The insects used in this work were fifth-instar nymphs, fasted for 12–15 days post-ecdysis. Rearing conditions were maintained at 28 ± 1 °C, 50–70% relative humidity, and a 12:12 h light—dark photoperiod. The insects were fed chicken blood once weekly.

### 2.3. Gas Chromatography (GC) Analyses of Terpenes

The extracts were analyzed by gas chromatography using a HS-20 headspace sampler and NEXIS GC-2030 chromatograph (Shimadzu, Japan) equipped with a fused silica column Rxi-624Sil MS (30 m × 0.25 mm, film thickness 1.4 μm (Restek, PA, USA)) and flame ionization detector. For the sample preparation, approximately 0.02 g of each sample was accurately weighed and directly sealed into a 20 mL (HS Headspace) vial. The HS operating conditions were as follows: static mode with loop (10 μL sample volume; 20 mL headspace vial) with equilibration time of 30 min; vial pressurization 1 min, equilibration 0.2 min; loop load time 1 min; and equilibration 0.2 min. The injection conditions were: time: 1 min; oven temperature: 150 °C; sample line temperature: 150 °C; transfer line temperature: 150 °C; and GC cycle time: 35 min. GC operating conditions were as follows: the carrier gas (helium) was set at a constant linear velocity of 47.2 cm/s and flow rate of 2.5 mL min^−1^; purge flow: 3 mL/min and pressure: 157.5 kPa; the split was 50.0; and the column temperature program of GC was initially set at 40 °C and held for 2 min before increasing at a rate of 10 °C/min to 150 °C, then kept for 1 min before being gradually increased to 250 °C (9 °C/min). Total GC run time: 35 min. The conditions applied for detection were temperature: 300 °C, H_2_ flowrate: 40 mL/min, make-up flowrate: 35 mL/min, and air flowrate: 400 mL/min. The quantification of components was performed with the help of a calibration curve (5 to 2000 ppm) using a standard mix of 19 terpenes (Restek Corporation, Bellefonte, PA, USA). Data acquisition and processing were controlled by LabSolutions software (version 5.73).

### 2.4. High-Performance Liquid Chromatography (HPLC) Analysis of Cannabinoids

The extracts were analyzed using a Shimadzu LC-2050 chromatograph-equipped UV–Vis detector (Kyoto, Japan) with a Raptor ARC-18 column (particle size: 5 µm, length: 250 mm, id: 4.6 mm, (Restek Corporation, Bellefonte, PA, USA). The injection volume was 10 µL. During analysis, the columns were maintained at 40 °C. Elution of the cannabinoids was monitored at 228 nm. Solvent A consisted of 0.1% (*v*/*v*) formic acid in 25 mM aqueous ammonium formate; mobile-phase solvent B was 0.1% (*v*/*v*) formic acid in acetonitrile. The elution was isocratic (mobile-phase A: mobile-phase B, 25:75). The HPLC flow rate was 1.5 mL/min and the run time 12.0 min. The quantification of components was performed with the help of a calibration curve (10 to 100 ppm) using a standard mix of 8 neutral cannabinoids, namely (-)-Δ^8^-THC, (-)-Δ^9^-THC, cannabidiol (CBD), cannabigerol (CBG), cannabinol (CBN), cannabichromene (CBC), cannabidivarin (CBDV), and tetrahydrocannabidivarin (THCV) (Cerilliant, TX, USA). Data acquisition and processing were controlled by Lab Solutions software (version 5.73) (Shimadzu, Kyoto, Japan).

### 2.5. Lethal Activity Against Insects by Direct Topical Application

**Figure 1:**
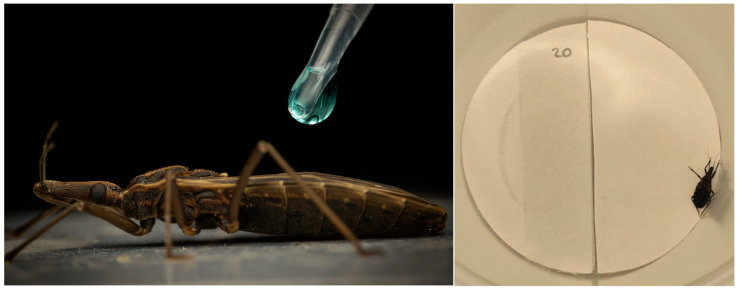
Topical application of treatment on a fifth-instar nymph of *T. infestans* (**left picture**). Setup for the contact bioassays to determine the lethal activity of treatments (**right picture**).

Topical and contact bioassays were performed to determine the direct lethal activity of extracts of *C. sativa* against fifth-instar nymphs of *T. infestans* [27] or adult workers of *A. mellifera*. In performing these we followed the protocol as described by [28]. In the topical assay, 1 µL of extract was applied to each nymph in the dorsal area of the abdomen using a 10 μL Hamilton syringe (Figure 1). The doses evaluated (0.5, 1, 5, 10, 20, 25, and 50 µg/insect for extracts) were obtained by dissolving the extracts with their corresponding solvent. Each dose of extract was evaluated in 10 insects (N = 3) and the effects of the treatment were evaluated at 24, 48, 72, and 96 h. Deltamethrin was used as a positive control for *T. infestans* and the neonicotinoid imidacloprid for *A. mellifera* (5, 10, 15, 20, 25, and 50 ng/insect). Control insects received 1 µL of pure solvent (acetone or ethanol) only.

### 2.6. Lethal Activity Against Insects by Surface Contact

Surface contact bioassays were performed to determine the lethal activity of extracts of *C. sativa* against fifth-instar nymphs of *T. infestans* [27]. The contact effect of the extracts was evaluated by treating 9 cm-diameter (64 cm^2^) Whatman No. 1 filter papers (Cytiva, Wilmington DE, USA). Dilutions of the extracts were made to test concentrations at 50, 100, 200, 400, 800, and 900 µg/cm^2^. Each filter paper was treated with 1 mL of dilutions; the paper’s surface was impregnated homogeneously using a pipette. After letting the papers evaporate for 24 h, 10 nymphs were placed on each filter paper for 1 h, then the nymphs were transferred to clean containers with folded papers and mortality was recorded at 24, 48, 72, and 96 h (N = 3). Control filter papers received 1 mL of pure acetone or ethanol only. Deltamethrin was used as a positive control at 0.2, 0.4, 0.8, and 1.5 µg/cm^2^. Nymphs unable to move from the center to the edge of the filter paper on their own, either spontaneously or after mechanical stimulation with a clamp, were considered dead.

### 2.7. Insect Repellent Activity Against Fifth-Instar Nymphs T. infestans

The repellent activity of the extracts was determined by the area preference test [10]. A 9 cm-diameter (64 cm^2^) Whatman No. 1 filter paper was divided into two halves; one of the halves was treated with 500 µL of the treatment (final concentrations of 12.5, 25, and 50 µg/cm^2^) while the other half received the same volume of vehicle (solvent). After allowing the solvents to evaporate for 24 h, the two halves of the paper were joined with adhesive tape and placed in Petri dishes (Ø 9 cm). One nymph was placed on each dish and its location on the paper recorded after 5, 15, 60, and 120 min. Control dishes contained one half of the paper with vehicle (solvent) and the other half not treated. The insect repellent N,N-Diethyl-meta-toluamide (DEET) was used as a positive control. We performed three independent experiments using 30 nymphs for each concentration of treatment or control. The extract’s repellent index (RI) was calculated using the formula: (number of insects in untreated half) − (number of insects in treated half)/(Total number of insects) × 100.

### 2.8. Combinatorial Treatments and Median-Effect Plot-Based Computational Analysis

We followed the method proposed by Chou-Talalay [29] which assumes that the dose–effect relationships of two drugs in single treatments and in combination are all parallel in the median-effect (EC50) plot. Synergy or antagonism between drugs deviate the plots away from this parallelism. The method calls, therefore, for the calculation of the median-effect (EC50) plot for each single-drug treatment and the combination treatment in a ratio (EC50drugA/EC50drugB) in different doses. From these data the fraction of affected insects at each dose (Fa) is calculated, and then the combination index (CI) and dose reduction index (DRI) values are calculated. The CI values < 1 indicate synergism, CI = 1 additivity, and CI > 1 antagonism, whereas DRI values > 1 indicate favorable dose reduction. We used Calcusyn^©^ (Biosoft, Cambridge, UK) to calculate the combination index (CI) and dose-reduction index (DRI) and generate the Fa–CI and DRI plots.

### 2.9. Statistical Analysis

Abbott’s formula was used to correct the mortality observed with each concentration or dose of the extracts by taking into account the mortality reported in the control nymphs [29]. For the determination of the parameters lethal dose 50% (LD 50%) (dose required to kill 50% of treated individuals), lethal concentration 50% (LC50) (concentration required to kill 50% of treated individuals), and 95% confidence intervals (CI 95%), the corrected data were analyzed with the Probit statistical model using the Polo-PC software program (LeOra Software, version 2.0) [30]. The statistical comparisons of LD50 and LC_50_ values among treatments were performed by examining the overlap of their 95% confidence intervals. Non-overlapping intervals were considered to indicate significant differences. To evaluate the repellent efficacy of the different compounds, the proportion of repelled nymphs was compared by a one-way analysis of variance for fixed effects with a pair-wise post hoc Tukey’s test. For repellency assays, RI values were compared among treatments and controls using one-way ANOVA followed by Tukey’s HSD post hoc test (*p* < 0.05).

## 3. Results

### 3.1. Plant Extracts Composition

The yield was 12% and 14% for the acetone and ethanol extract, respectively. The small difference in polarity of the solvents led to great differences in the presence and concentration of terpenes (Table 1) and cannabinoids (Table 2) between the two extracts. Acetone extract was superior to ethanol extract in the concentration of terpenes and cannabinoids. Only 10 of the 19 analyzed terpenes were successfully identified and quantified in the extracts. The sesquiterpene *β*-caryophyllene was the most abundant, with a concentration four times higher in the acetone extract (418 ppm vs. 138 ppm), followed by the monoterpenes *β*-pinene (149 ppm), *d*-limonene (178 ppm), *E*-ocimene (278 ppm), and the sesquiterpene *α*-humulene (140 ppm), while in the ethanol extract the isomer *β*-pinene was the only terpene that exceeded 100 ppm.

In terms of cannabinoids, cannabidiol (CBD) and Δ^9^-tetrahydrocannabinol (Δ^9^-THC) were the most abundant cannabinoids in the two extracts, and the relationship between the two cannabinoids (Δ^9^-THC/CBD) was 1.1:1 and 1.4:1 for the ethanol and acetone extracts, respectively.

### 3.2. Lethal Activity of C. sativa Extracts and Its Main Volatile Components in Single Treatments Against Late-Stage Nymphs of T. infestans

The lethal effect of the extracts was not only evaluated considering their potency, but also the record of their effect throughout the post-contact time. For the topical assay, we recorded the maximum activity of the two extracts 48 h after application to the nymphs. The highest potency with respect to the lethal effect of 50% was registered by the acetone extract, which was five times more potent than the ethanol extract. The lethal activity of deltamethrin was 23 and 121 times more potent than the acetone and ethanol, respectively. Among the four terpenes tested, *β*-caryophyllene exhibited the highest toxicity, with LD_50_ values of 51.7 µg/individual at 24 h and 31.4 µg/individual at 48 h. d-Limonene showed intermediate toxicity (LD_50_ = 68.5 and 51.4 µg/individual at 24 h and 48 h, respectively), followed by *β*-pinene and *E*-ocimene, which were the least toxic compounds. *E*-ocimene displayed the highest LD_50_ values, with 129.4 µg/individual at 24 h and 96.3 µg/individual at 48 h, indicating a comparatively weak insecticidal effect. As observed for the extracts, all terpenes reached their maximum lethal effect at 48 h. When compared to the complete extracts, all individual terpenes exhibited notably lower toxicity. At 48 h, AE (the most potent treatment) was approximately 15, 24, 32, and 46 times more potent than *β*-caryophyllene, *d*-limonene, *β*-pinene, and *E*-ocimene, respectively (Table 3).

Regarding the determination of the lethal concentrations 50% (contact test on filter paper), we recorded a pattern of toxicity similar to that observed in the topical test (Table 4). The maximum lethal activity was observed 48 h after the first contact of the insects with the disk papers, and, in addition, the acetone extract was seven times more potent than the ethanol extract. In this assay the difference in lethal potency between deltamethrin and the extracts was greater than that observed in the topical assay. The lethal potency of deltamethrin was 90 and 617 times that of the acetone and ethanol extract, respectively.

### 3.3. Insect Repellent Activity Against Fifth-Instar Nymphs of T. infestans

**Figure 2:**
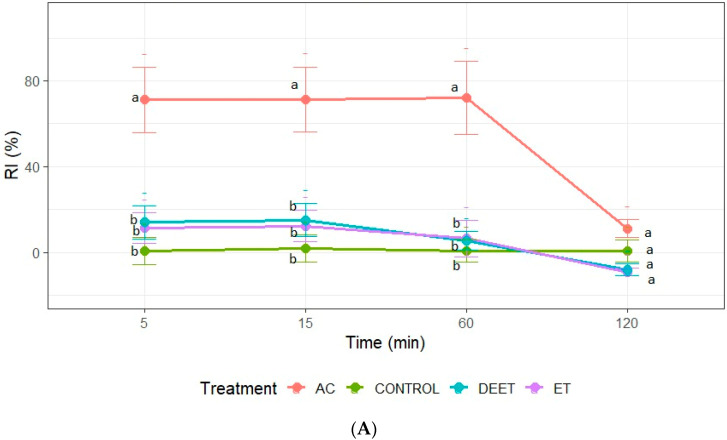

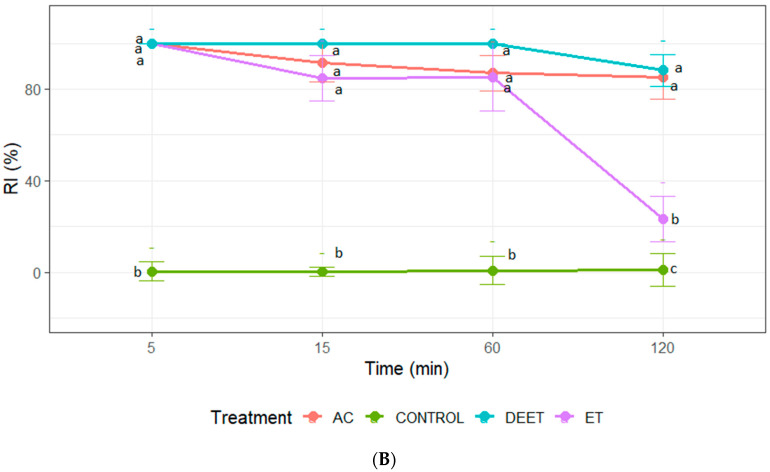
Repellence index (RI) of *C. sativa* extracts (AC: acetone extract; ET: ethanol extract), control (acetone or ethanol), and positive control (DEET) at 25 µg/cm^2^ (**A**); and (**B**) 50 µg/cm^2^ against fifth-instar nymphs of *T. infestans*. Bars express 95% confidence intervals. Different letters indicate statistically significant differences (*p* < 0.05; non-overlapping 95% confidence intervals).

Repellence was recorded only at concentrations of 25 and 50 µg/cm^2^. At the maximum concentration, acetone and DEET achieved a similar repellent effect with values close to 100% at all evaluation times. In the case of ethanol extract, its effect was similar to acetone extract and DEET only at 5, 15, and 60 min, losing its repellent effect after 120 min of exposure to insects (Figure 2). It should be noted that only acetone extract demonstrated a repellent effect on the nymphs at the concentration of 25 µg/cm^2^. The repellent activity of the extract was recorded during the first 60 min of exposure. Statistical analysis confirmed that the RI values of the acetone extract were significantly higher than those of the ethanol extract and the negative control (ANOVA, *p* < 0.05; Tukey’s test) (Figure 2).

### 3.4. Toxicity Against Adult A. mellifera

The extracts of *C. sativa* did not demonstrate toxicity against the beneficial insect *Apis mellifera*, i.e., <10% mortality at 96 h at all doses evaluated. The LD50 of the neonicotinoid imidacloprid, used as a positive control during the assay, was 14.3 ng/bee.

### 3.5. Analysis of Synergies in Combination Treatments

The extracts were combined in binary mixtures with their three most abundant terpenes. In the case of the AE, these were *β*-caryophyllene, *E*-ocimene, and *d*-limonene, while for the ethanolic extract (EE), they were *β*-caryophyllene, *β*-pinene, and *d*-limonene. The ratio for each combination treatment was determined by the simpler fraction of the coefficient between LD50s (µg/insect) at 48 h (Table 3). For example, the combination treatment AE + *β*-caryophyllene was tested at LD50(AE)/LD50(*β*-caryophyllene) = 2.1/31.4 @ 1/15. The LD50s of these combined doses are presented in Table 5. The results of the application of several doses of the combination treatments led to the calculation of their potential synergies by the Chou-Talalay method, and these are summarized in Table 6.

The binary combinations of *C. sativa* extracts with their most abundant terpenes revealed diverse interaction patterns against late-stage nymphs of *T. infestans* at 48 h post-treatment. The combination of AE with *β*-caryophyllene (1:15) demonstrated strong synergistic activity. At 8 µg × insect, this mixture caused 85% mortality with a CI of 0.17, and a DRI of 18.1 for AE and 8.4 for *β*-caryophyllene. A lower CI value was achieved at 16 µg × insect (CI = 0.06), with 99% mortality and even higher DRIs (AE: 106.4; *β*-caryophyllene: 17.1), confirming a robust synergistic effect (Table 6). In contrast, the AE + *E*-ocimene (1:48) mixture showed variable effects depending on the dose. At 17.5 µg, a near-additive interaction was observed (CI = 1.10; DRI AE = 1.4; DRI *E*-ocimene = 1.8). However, at higher doses (35–280 µg) the interaction became antagonistic, with CI values increasing from 1.60 to 4.80 and DRI values decreasing below 1 (e.g., DRI AE = 0.4–1.1; DRI EO = 0.3–1.3), indicating reduced efficacy of the combination at increased concentrations. The AE + *d*-limonene (1:25) combination was synergistic at 26 µg (91% mortality, CI = 0.22), with DRI values of 11.4 for AE and 7.4 for *d*-limonene. A moderate synergistic effect was also noted at 13 µg (CI = 0.58; DRI AE = 3.3; DRI DL = 3.1) (Table 6).

For the EE, the combination with *β*-caryophyllene (1:3) displayed synergy at 20 µg (CI = 0.27), with DRI values of 12.9 for EE and 5.1 for the terpene. A moderate synergistic effect was also observed at 10 µg (CI = 0.57; DRI EE = 3.6; DRI BC = 3.1). The EE + *d*-limonene (1:5) mixture showed consistent synergistic interactions across multiple doses. CI values ranged from 0.32 to 0.57 (for 15–60 µg), and the DRI values for EE ranged from 3.7 to 6.7, while d-limonene exhibited DRI values between 3.4 and 4.6. On the other hand, EE + *β*-pinene (1:7) was largely antagonistic. CI values increased progressively with dose (2.80 to 9.00), and DRI values for *β*-pinene remained low (0.2–1.28), suggesting an inefficient combination (Table 6).

**Figure 3:**
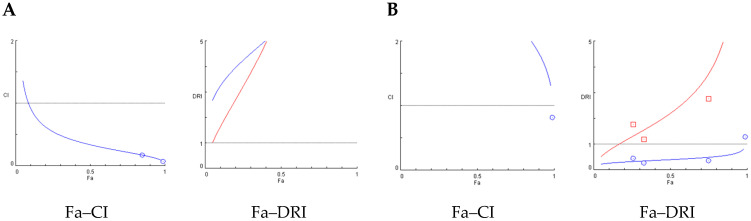
Chou-Talalay (Fa–CI) and the Fa–Chou–Martin DRI plot (Fa–DRI) for constant ratio combination treatments. Blue curves correspond to the extracts alone, while red curves represent the combination treatments Left: Fa–CI plots, where Fa is the fraction affected and CI the combination index (CI < 1 indicates synergism, CI = 1 additivity, CI > 1 antagonism). Right: Fa–DRI plots, where DRI is the dose reduction index (values > 1 indicate favorable dose reduction). (**A**) Fa–CI and Fa–DRI plots for the AE and β-caryophyllene combination at 48 h; (**B**) Fa–CI and Fa–DRI plots for the AE + E-ocimene combination at 48 h.

Figure 3 shows the Fa–CI and Fa–DRI plots. In these graphs, Fa represents the fraction of affected insects at each dose, CI corresponds to the combination index, and DRI to the dose reduction index. CI values < 1 indicate synergism, CI = 1 additivity, and CI > 1 antagonism, whereas DRI values > 1 indicate favorable dose reduction. These parameters allow visualization of how the interaction between extract and terpene varies across different effect levels. For the combination of AE and β-caryophyllene, generated using the Chou-Talalay method. The Fa–CI plot (left) shows CI values consistently below 1 across the entire effect range (Fa), indicating a clear synergistic interaction between the components. Notably, CI values decrease as Fa increases, suggesting stronger synergy at higher effect levels. The corresponding Fa–DRI plot (right) reveals DRI values greater than 1 for both compounds, indicating that their doses can be significantly reduced when used in combination to achieve the same biological effect (Figure 3A). The Fa–CI and Fa–DRI plots for the AE + *E*-ocimene combination at 48 h (Figure 3B) reveal a distinct antagonistic profile. As shown in the Fa–CI plot, CI values remain consistently above 1, particularly at high effect levels (Fa > 0.7), indicating antagonism between the two components. In line with this, the Fa–DRI plot demonstrates that the DRI for both AE and *E*-ocimene remains below or close to 1 across most of the effect range.

## 4. Discussion

Due to the increasing number of reports of *T. infestans* populations resistant to the primary insecticides used for their control, along with the limited investment in developing new replacements, vector control programs face a significant challenge [1,2]. The urgent need for eco-friendly products to manage disease vectors is well-recognized [30]. This study represents the first report on the lethal and repellent effects of *C. sativa* inflorescence extracts on late-stage nymphs of *T. infestans*. It should be noted that the present assays were performed on a long-term laboratory colony of *T. infestans* that has been maintained without insecticide exposure since 2010. As such, these insects are expected to be highly susceptible compared to many field populations, which can be highly resistant to pyrethroids. This limitation should be considered when extrapolating the present results to field conditions.

Our extracts consist of volatile components, such as monoterpenes and small phenolic compounds, as well as phytocannabinoids (meroterpenes that are part terpenes, part phenols). These classes of compounds are increasingly considered environmentally friendly options in the field of pesticide development [31,32]. Also important is the choice of solvent, with simple alcohols (e.g., methanol, ethanol) and alkanes (e.g., heptane, hexane) generally considered environmentally preferable solvents [33]. Acetone, however, is also an acceptable option, along with 1-butanol, 2-propanol, and glycerol, as these bio-renewable alternatives do not generate harmful by-products [34]. We tested ethanol and acetone as extraction solvents. The higher concentration of phytochemicals in the acetone extract correlated with a superior bioactivity in all assays as well as an absence of toxicity towards the beneficial insect *Apis mellifera*.

Previous studies have documented the insecticidal activity of *C. sativa* extracts. For instance, acetone and methanol extracts of *C. sativa* at concentrations of 5, 10, and 20% caused 100% mortality in adults of the economically damaging pulse beetle *Callosobruchus chinensis* [35]. Research on the lethal and repellent effects of *C. sativa* extracts has predominantly focused on mosquito species. For example, Jalees et al. demonstrated the larvicidal activity of an ethanol extract of *C. sativa* leaves, reporting LC50 values of approximately 1000, 1400, and 5000 mg/L for *Anopheles stephensi*, *Culex quinquefasciatus*, and *Aedes aegypti*, respectively [21], which are vectors of malaria, filariasis, and dengue/Zika/yellow fever, respectively.

In our study, the maximum lethality of *T. infestans* nymphs occurred 48 h post-contact with the extracts. A similar delayed lethal effect of *C. sativa* extracts was observed for *Culex quinquefasciatus* [36] and *Anopheles stephensi* [37]. The time required for an insecticide to reach its lethal concentration in an insect determines whether it causes rapid mortality (less than 24 h) or delayed mortality (over 24 h) [38]. In *T. infestans* nymphs, the first signs of intoxication from *C. sativa* extracts included a significant reduction in mobility 24 h post-contact, followed by incoordination and eventual death at 48 h. These signs differ from those caused by the neuroinsecticide deltamethrin, a fast-acting insecticide that induces hyperexcitation, incoordination, and death. Intoxication symptoms are directly linked to the modes of action of insecticides. For essential oils and extracts of *C. sativa*, proposed mechanisms include inhibition of the enzyme acetylcholinesterase and interactions with octopaminergic, gabaergic, and cholinergic receptors [20]. Further research is needed to elucidate the specific mechanisms responsible for the delayed mortality observed in this study.

The insecticidal effect of many plant volatiles is well-known and ubiquitously present in the literature. Therefore, we were interested in whether “fortifying” the extract with known amounts of individually isolated, commercially affordable terpenes would result in a combinatorial positive effect. The choice of four terpenes was guided by a targeted analysis within a set of 19 known terpenes shortlisted by Restek as commonly present in *Cannabis* sp. Only a subset of 10 of these VOCs listed in Table 1 could be detected and quantified in our S/L extracts. While they are not an exhaustive list of all possible main or minor VOCs in the original plant material, they became the focus of our study. Therefore, *β*-caryophyllene, *β*-pinene, and *d*-limonene were selected. Indeed they are responsible for some of the most mainstream cannabis chemotypes, as per Hanuš & Hod [39], and are reported as having promising insecticide activity, as discussed below and more generally by Liu and coworkers [40]. Surprisingly, *E*-ocimene, which is also present in several *Cannabis* chemotypes as a minor component [39], as well as being endowed with general insecticide activity [41] was one of the four top terpenes identified. Interestingly, there are reports of its role on the beneficial insect *A. mellifera* [42]. It appears that this volatile is synthesized by bees to stimulate pollen foraging among other functions. Therefore, it was also interesting to see its selective toxicity on both the bee workers and the vector’s nymphs.

The remaining terpenes identified in our *Cannabis* extracts (Linalool, α-Humulene, Nerolidol, (-)-Guaiol, and (-)-α-Bisabolol) are also mentioned in the literature as having insecticide effects [40,43,44,45,46]. However, they turned out to be less abundant in our extracts and were not selected for further bioassays as we could not possibly test all of them due to time and budget constraints and insect availability.

The difference in lethal potency between the acetone and ethanol extracts of *C. sativa* may be attributed to the concentration of toxic molecules present. For example, the sesquiterpene *β*-caryophyllene, which is four times more concentrated in the acetone extract, has been identified as a major constituent in a *C. sativa* essential oil with toxic activity against *Aedes albopictus* larvae and *Aedes aegypti* larvae and adults [47,48]. In triatomines, *β*-caryophyllene applied topically demonstrated lethality against nymphs (stages I to V) and adults of *Meccus pallidipennis* and *Meccus bassolsae*, two vectors of *T. cruzi* in Mexico [49]. Consistent with these findings, our bioassays revealed that β-caryophyllene produced strong synergistic interactions when combined with both acetone and ethanol extracts of *C. sativa*, markedly increasing their lethal potency. The combination of *β*-caryophyllene with the acetone extract exhibited robust synergy, with combination index (CI) values as low as 0.06–0.17, reflecting a significant enhancement in insecticidal efficacy and a substantial dose reduction and highlighting the importance of this terpene as a key modulator of extract bioactivity. Meanwhile, synergy with the ethanol extract was also observed but to a lesser extent, demonstrating how extract composition influences these interactions.

Limonene was the second most abundant monoterpene in our extracts. Previous research has shown that high concentrations of limonene in *C. sativa* essential oil are associated with larvicidal efficacy against *A. aegypti* [48]. In our study, binary mixtures of limonene with both acetone and ethanol extracts produced strong synergism (CI values < 0.2, with high dose-reduction indices), markedly enhancing the lethality of the extracts at lower concentrations. Such potentiation is consistent with previous reports indicating that *d*-limonene can synergize due to its superior cuticular penetration and solubility, as well as its ability to operate via multiple modes of action—including contact toxicity, feeding deterrence, and fumigant activity—when combined with other natural compounds [50].

Other monoterpenes present in the acetone extract such as linalool and nerolidol are able to disrupt the cuticle of *T. infestans* [51]. The potency of the acetone extract may also be due to its increased cannabinoid concentration. For instance, Mantzoukas et al. reported dose-dependent pesticide activity of commercial CBD oil against larvae of *Tribolium confusum*, *Oryzaephilus surinamensis*, and *Plodia interpunctella*, achieving 100% lethality at the highest dose (90 mg/mL) [52].

Interestingly, the presence of cannabinoid receptors in insects remains elusive. McPartland et al. were unable to identify orthologs of human CB receptors in the *Drosophila* genome, suggesting that cannabinoid effects in insects are mediated through CB-independent mechanisms [53]. Conversely, nicotinic acetylcholine receptors (nAChRs), which are targeted by nicotine and its analogs, are present in both humans and insects [54]. Research on the insecticidal activities of phytocannabinoids have recently shown that the chewing herbivore *Trichoplusia ni* larvae grew less and had lower rates of survival when their diets contained cannabidiol acid and cannabigerol acid from hemp [55] and that the CBD-rich methanol fraction of hemp leaf extracts induced concentration-dependent larvicidal activity against mosquito larvae from both pyrethroid-susceptible and pyrethroid-resistant strains of *Aedes aegypti* [56]. The defensive role of CBD was demonstrated with tobacco hornworm *Manduca sexta* [57]. However, evidence about the effect of other phytocannabinoids and their mechanisms of action are not yet well characterized.

Considering the findings presented here, the ultrasound-assisted acetone extract exerts acute toxicity to *T. infestans*—a vector of major public health implications—while not to honeybees—a model of a beneficial insect. Importantly, our bioassays also revealed strong synergistic interactions when extracts were combined with *β*-caryophyllene and *d*-limonene, which significantly enhanced insecticidal potency at lower doses. These results highlight the relevance of terpene–extract interactions in shaping the overall efficacy of *C. sativa*-based bio-insecticides. Future studies should focus on elucidating the mechanisms of these effects and how the main terpenes and cannabinoids contribute to the overall insecticidal activity at a mechanistic level. Outstanding questions such as demonstrating control at the population level would require considerable field trials. Similarly, extending the study to other economically and ecologically important insects would be important to predict nontarget effects in the field, including sublethal effects on honeybees.

## Figures and Tables

**Table 1:** Gas chromatography analysis of terpenes (ppm) present in *C. sativa* extracts.

Compound	Ethanol Extract	Acetone Extract
*α*-Pinene	<25	36.6
*β*-Pinene	105.6	149.8
*d*-Limonene	55.1	178.4
*E*-Ocimene	51.2	278.5
Linalool	<25	60.3
*β*-Caryophyllene	139.9	418.2
*α*-Humulene	50.1	141.0
Nerolidol	<25	64.6
(-)-Guaiol	<25	<25
(-)-*α*-Bisabolol	<25	95.5

**Table 2:** High-performance liquid chromatography analysis of cannabinoids (mg/mL) present in *C. sativa* extracts.

Compound	Ethanol Extract	Acetone Extract
Cannabidivarin (CBDV)	-	<1
Cannabigerol (CBG)	-	<1
Cannabidiol (CBD)	1.8	3.8
Tetrahydrocannabivarin (THCV)	-	-
Cannabinol (CBN)	-	<1
Δ^9^-tetrahydrocannabinol (THC)	2.0	5.4
Cannabichromene (CBC)	0.6	<1

**Table 3:** Determination of the topical lethal dose 50% (LD50) of *C. sativa* extracts and its main volatile components against late-stage nymphs of *T. infestans*. A total of 24 and 48 h post-administration (N = 3). Values are expressed with 95% confidence intervals. Statistical differences between treatments were determined based on non-overlapping confidence intervals. Different superscript letters indicate statistically significant differences (*p* < 0.05; non-overlapping 95% confidence intervals).

Treatment	LD 50% (µg/Individual) (CI 95%)	
	24 h	48 h
Deltamethrin	0.09 (0.03–0.15) ^a^	0.09 (0.03–0.15) ^a^
Acetone extract (AE)	5.1 (3.8–11.2) ^b^	2.1 (1.4–2.9) ^b^
Ethanol extract (EE)	13.9 (9.2–43.3) ^bc^	10.9 (7.5–26.2) ^bc^
*β*-caryophyllene	51.7 (38.7–75.6) ^c^	31.4 (18.1–59.8) ^c^
*d*-limonene	68.5 (52.3–93.6) ^cd^	51.4 (37.9–68.5) ^cd^
*β*-pinene	92.2 (74.7–115.2) ^de^	68.3 (52.7–92.1) ^de^
*E*-ocimene	129.4 (99.3–184.4) ^e^	96.3 (75.2–126.2) ^e^

**Table 4:** Determination of the lethal concentrations 50% (LC50) by surface contact exposure in fifth-instar nymphs of *T. infestans* at 24 and 48 h post-contact (N = 3). Values are expressed with 95% confidence intervals. Statistical differences between treatments were determined based on non-overlapping confidence intervals. Different superscript letters indicate statistically significant differences (*p* < 0.05; non-overlapping 95% confidence intervals).

Treatment	LD 50% (µg/cm^2^) (CI 95%)	
	24 h	48 h
Acetone extract	1.3 (0.9–1.7) ^a^	1.3 (0.9–1.7) ^a^
Ethanol extract	212.4 (150.3–332.5) ^b^	117.1 (45.9–321.8) ^b^
Deltamethrin	998.1 (564.3–1322) ^c^	803.7 (555–1324.5) ^c^

**Table 5:** Determination of the topical lethal dose 50% (LD50) of combination treatments of *C. sativa* extracts with its main volatile components against late-stage nymphs of *T. infestans* at 24 h and 48 h post-administration. Different superscript letters indicate statistically significant differences (*p* < 0.05; non-overlapping 95% confidence intervals).

Combination Treatment (Ratio)	LD 50% (µg/Insect) (CI 95%)	
	24 h	48 h
AE + *β*-caryophyllene (1:15)	6.1 (3.1–24.8) ^a^	5.2 (3.2–12.3) ^a^
AE + *d*-limonene (1:25)	18.1 (5.2–44.3) ^a^	14.1 (4.3–31.5) ^a^
EE + *β*-caryophyllene (1:3)	12.3 (9.6–17.3) ^a^	10.6 (6.3–13.3) ^a^
EE + *d*-limonene (1:5)	19.2 (13.2–28.9) ^a^	15.9 (7.3–33.9) ^a^
AE + *E*-ocimene (1:48)	135.3 (74.5–165.4) ^b^	108.4 (75.2–125.9) ^b^
EE + *β*-pinene (1:7)	287.2 (111.3–369.3) ^bc^	240.3 (156.9–301.3) ^c^

**Table 6:** Determination of the topical lethal dose 50% (LD50) of combination treatments of *C. sativa* extracts with its main volatile components against late-stage nymphs of *T. infestans* at 48 h post-administration.

Combination Treatment	Dose (µg)	% Death	CI	Effect	DRI AE	DRI EE	DRI BC	DRI BP	DRI DL	DRI EO
AE + *β*-caryophyllene (1:15)	8	85	0.17	S	18.1	-	8.4	-	-	-
	16	99	0.06	S	106.4	-	17.1	-	-	-
AE + *E*-ocimene (1:48)	17.5	23	1.10	+	1.4	-	-	1.8	-	-
	35	34	1.60	A	1.1	-	-	1.3	-	-
	70	39	2.70	A	0.7	-	-	0.7	-	-
	140	55	3.30	A	0.6	-	-	0.5	-	-
	280	66	4.80	A	0.4	-	-	0.3	-	-
AE + *d*-limonene (1:25)	13	44	0.58	S	3.3	-	-	-	3.1	-
	26	91	0.22	S	11.4	-	-	-	7.4	-
EE + *β*-caryophyllene (1:3)	10	45	0.57	S	-	3.6	3.1	-	-	-
	20	89	0.27	S	-	12.9	5.1	-	-	-
EE + *d*-limonene (1:5)	15	44	0.57	S	-	3.7	-	-	3.4	-
	30	79	0.32	S	-	6.7	-	-	4.6	-
	60	89	0.42	S	-	6.4	-	-	3.7	-
EE + *β*-pinene (1:7)	112.5	26	2.80	A	-	1.7	-	-	-	0.4
	245	33	4.60	A	-	1.1	-	-	-	0.2
	490	75	3.10	A	-	1.7	-	-	-	0.3
	980	99	0.80	S	-	27.5	-	-	-	1.28

Effect codes: (S) synergism (CI 0.1–0.90); (+) additive (CI = 0.90–1.1); (A) antagonism (CI = 1.10–10).

## Data Availability

All data are available either in the manuscript or in the Supplementary Materials.

## Supplementary Materials

The following supporting information can be downloaded at: https://www.mdpi.com/article/10.3390/plants14213258/s1.

## Abbreviations

The following abbreviations are used in this manuscript:

CB	Cannabinoid
CBC	Cannabicromeno
CBD	Cannabidiol
CBDV	Cannabidivarin
CBG	Cannabigerol
CBN	Cannabinol
DEET	N,N-Diethyl-meta-toluamide
GC	Gas chromatography
LC_50_	Lethal concentration 50%
LD_50_	Lethal dose 50%
HPLC	High-performance liquid chromatography
nAChRs	Nicotinic acetylcholine receptors
THC	Δ^9^-Tetrahydrocannabinol
THCV	Tetrahydrocannabivarin
UAE	Ultrasound-assisted extraction
UV	Ultraviolet

## References

[1] Devine G.J., Overgaard H.J., Paul R.E. (2019). Global Vector Control Guidelines—The Need For Co-Creation. Trends Parasitol..

[2] Mougabure-Cueto G., Picollo M.I. (2015). Insecticide Resistance in Vector Chagas Disease: Evolution, Mechanisms and Management. Acta Trop..

[3] WHO (2025). Chagas Disease (Also Known as American Trypanosomiasis).

[4] Pessoa G.C.D., Vinãs P.A., Rosa A.C.L., Diotaiuti L. (2015). History of Insecticide Resistance of Triatominae Vectors. Rev. Soc. Bras. Med. Trop..

[5] Carvajal G., Picollo M.I., Toloza A.C. (2014). Is Imidacloprid an Effective Alternative for Controlling Pyrethroid-Resistant Populations of *Triatoma infestans* (Hemiptera: Reduviidae) in the Gran Chaco Ecoregion?. Mem. Inst. Oswaldo Cruz.

[6] Alarico A.G., Romero N., Hernández L., Catalá S., Gorla D. (2010). Residual Effect of a Micro-Encapsulated Formulation of Organophosphates and Piriproxifen on the Mortality of Deltamethrin Resistant *Triatoma infestans* Populations in Rural Houses of the Bolivian Chaco Region. Mem. Inst. Oswaldo Cruz.

[7] Dadé M.M., Daniele M.R., Silvestrini M.P., Bozzolo F., Francini F., Mestorino N. (2020). A Study of the Effects of Imidacloprid under Laboratory and Field Conditions on Nymphs of *Triatoma infestans* (Hemiptera: Reduviidae). Vet. Parasitol..

[8] Dadé M.M., Daniele M.R., Machicote M., Errecalde J.O., Rodriguez-Vivas R.I. (2020). First Report of the Lethal Activity and Synergism between Deltamethrin, Amitraz and Piperonyl Butoxide against Susceptible and Pyrethroid-Resistant Nymphs of *Triatoma infestans*. Exp. Parasitol..

[9] Ferrero A.A., Werdin González J.O., Sánchez Chopa C. (2006). Biological Activity of *Schinus molle* on *Triatoma infestans*. Fitoterapia.

[10] Dadé M., Zeinsteger P., Bozzolo F., Mestorino N. (2018). Repellent and Lethal Activities of Extracts From Fruits of Chinaberry (*Melia azedarach* L., Meliaceae) Against *Triatoma infestans*. Front. Vet. Sci..

[11] Mojica M., Alzogaray R.A., Mengoni S.L., Reynoso M.M.N., Pinto C.F., Niemeyer H.M., Echeverría J. (2020). Repellent Activity of the Essential Oil from *Laurelia sempervirens* (Ruiz & Pav.) Tul. (Monimiaceae) on *Triatoma infestans* (Klug) (Reduviidae). Bol. Latinoam. Caribe Plantas Med. Aromat..

[12] Moretti A.N., Seccacini E.A., Zerba E.N., Canale D., Alzogaray R.A. (2017). The Botanical Monoterpenes Linalool and Eugenol Flush-Out Nymphs of *Triatoma infestans* (Hemiptera: Reduviidae). J. Med. Entomol..

[13] Li H.-L. (1974). The Origin and Use of Cannabis in Eastern Asia Linguistic-Cultural Implications. Econ. Bot..

[14] Flores-Sanchez I.J., Verpoorte R. (2008). Secondary Metabolism in Cannabis. Phytochem. Rev..

[15] Kinghorn A.D., Falk H., Gibbons S., Kobayashi J. (2018). Phytocannabinoids: Unraveling the Complex Chemistry and Pharmacology of Cannabis sativa.

[16] Russo E.B., Pertwee R. (2016). The Pharmacological History of Cannabis. Handbook of Cannabis.

[17] Groeneveld G.J., Martin J.H. (2020). Parasitic Pharmacology: A Plausible Mechanism of Action for Cannabidiol. Br. J. Clin. Pharmacol..

[18] Liktor-Busa E., Keresztes A., LaVigne J., Streicher J.M., Largent-Milnes T.M. (2021). Analgesic Potential of Terpenes Derived from *Cannabis sativa*. Pharmacol. Rev..

[19] Abe H., Dadji G.A.F., Nkondjio C.A., Awono-Ambene P.H., Tamesse J.L. (2018). Insecticidal Activity of *Cannabis sativa* L Leaf Essential Oil on the Malaria Vector *Anopheles gambiae* s.l (Giles). Int. J. Mosq. Res..

[20] Benelli G., Pavela R., Petrelli R., Cappellacci L., Santini G., Fiorini D., Sut S., Dall’Acqua S., Canale A., Maggi F. (2018). The Essential Oil from Industrial Hemp (*Cannabis sativa* L.) by-Products as an Effective Tool for Insect Pest Management in Organic Crops. Ind. Crops Prod..

[21] Jalees S., Sharma S.K., Rahman S.J., Verghese T. (1993). Evaluation of Insecticidal Properties of an Indigenous Plant, *Cannabis sativa* Linn., against Mosquito Larvae under Laboratory Conditions. J. Entomol. Res..

[22] Stefkov G., Cvetkovikj Karanfilova I., Stoilkovska Gjorgievska V., Trajkovska A., Geskovski N., Karapandzova M., Kulevanova S. (2022). Analytical Techniques for Phytocannabinoid Profiling of Cannabis and Cannabis-Based Products—A Comprehensive Review. Molecules.

[23] Andre C.M., Hausman J.-F., Guerriero G. (2016). *Cannabis sativa*: The Plant of the Thousand and One Molecules. Front. Plant Sci..

[24] Aliferis K.A., Bernard-Perron D. (2020). Cannabinomics: Application of Metabolomics in Cannabis (*Cannabis sativa* L.) Research and Development. Front. Plant Sci..

[25] Jadhav P.D., Shim Y.Y., Paek O.J., Jeon J.-T., Park H.-J., Park I., Park E.-S., Kim Y.J., Reaney M.J.T. (2023). A Metabolomics and Big Data Approach to Cannabis Authenticity (Authentomics). Int. J. Mol. Sci..

[26] Das P.C., Vista A.R., Tabil L.G., Baik O.-D. (2022). Postharvest Operations of Cannabis and Their Effect on Cannabinoid Content: A Review. Bioengineering.

[27] WHO (1994). Protocolo de evaluation de efecto insecticida sobre triatominos. Acta Toxicol. Argent..

[28] Suchail S., Guez D., Belzunces L.P. (2000). Characteristics of Imidacloprid Toxicity in Two *Apis mellifera* Subspecies. Environ. Toxicol. Chem..

[29] Chou T.-C., Talalay P. (1984). Quantitative Analysis of Dose-Effect Relationships: The Combined Effects of Multiple Drugs or Enzyme Inhibitors. Adv. Enzym. Regul..

[30] Report of The16th FAO/WHO Joint Meeting on Pesticide Management: Geneva, Switzerland and Online, 6–10 November 2023. https://www.who.int/publications/i/item/9789240089808.

[31] Kumar S., Mahapatro G.K., Yadav D.K., Tripathi K., Koli P., Kaushik P., Sharma K., Nebapure S. (2022). Essential Oils as Green Pesticides: An Overview. Indian J. Agric. Sci..

[32] Ona G., Balant M., Bouso J.C., Gras A., Vallès J., Vitales D., Garnatje T. (2022). The Use of *Cannabis sativa* L. for Pest Control: From the Ethnobotanical Knowledge to a Systematic Review of Experimental Studies. Cannabis Cannabinoid Res..

[33] Capello C., Fischer U., Hungerbühler K. (2007). What Is a Green Solvent? A Comprehensive Framework for the Environmental Assessment of Solvents. Green Chem..

[34] Biorenewable Solvents. https://www.sigmaaldrich.com/GB/en/campaigns/biorenewable-solvents.

[35] Thakur D., Devi B. (2016). Biopesticidal Efficacy of Berberis *Lycium* L. and *Cannabis sativa* L. against *Callosobruchus chinensis* L (1758) (Coleoptera: Bruchidae). J. Insect Sci..

[36] Maurya P., Mohan L., Sharma P., Srivastava C.N. (2008). Larval Susceptibility of *Aloe barbadensis* and *Cannabis sativa* against *Culex quinquefasciatus*, the Filariasis Vector. J. Environ. Biol..

[37] Maurya P., Mohan L., Sharma P., Batabyal L., Srivastava C.N. (2007). Larvicidal Efficacy of *Aloe barbadensis* and *Cannabis sativa* against the Malaria Vector *Anopheles stephensi* (Diptera: Culicidae). Entomol. Res..

[38] Busvine J.R. (1971). A Critical Review of the Techniques for Testing Insecticides.

[39] Hanuš L.O., Hod Y. (2020). Terpenes/Terpenoids in Cannabis: Are They Important?. Med. Cannabis Cannabinoids.

[40] Liu Z., Li Q.X., Song B. (2022). Pesticidal Activity and Mode of Action of Monoterpenes. J. Agric. Food Chem..

[41] Ogendo J.O., Kostyukovsky M., Ravid U., Matasyoh J.C., Deng A.L., Omolo E.O., Kariuki S.T., Shaaya E. (2008). Bioactivity of *Ocimum gratissimum* L. Oil and Two of Its Constituents against Five Insect Pests Attacking Stored Food Products. J. Stored Prod. Res..

[42] Maisonnasse A., Lenoir J.-C., Beslay D., Crauser D., Le Conte Y., Giurfa M. (2010). E-Beta-Ocimene, a Volatile Brood Pheromone Involved in Social Regulation in the Honey Bee Colony (*Apis mellifera*). PLoS ONE.

[43] Liu T., Wang C.-J., Xie H.-Q., Mu Q. (2013). Guaiol—A Naturally Occurring Insecticidal Sesquiterpene. Nat. Prod. Commun..

[44] Al-Ghanim K.A., Krishnappa K., Pandiyan J., Nicoletti M., Gurunathan B., Govindarajan M., Al-Ghanim K.A., Krishnappa K., Pandiyan J., Nicoletti M. (2023). Insecticidal Potential of *Matricaria chamomilla*’s Essential Oil and Its Components (E)-β-Farnesene, Germacrene D, and α-Bisabolol Oxide A against Agricultural Pests, Malaria, and Zika Virus Vectors. Agriculture.

[45] Benelli G., Pavela R., Drenaggi E., Desneux N., Maggi F. (2020). Phytol, (*E*)-Nerolidol and Spathulenol from *Stevia Rebaudiana* Leaf Essential Oil as Effective and Eco-Friendly Botanical Insecticides against *Metopolophium dirhodum*. Ind. Crops Prod..

[46] Rants’o T.A., Koekemoer L.L., van Zyl R.L. (2023). The Insecticidal Activity of Essential Oil Constituents against Pyrethroid-Resistant *Anopheles funestus* (Diptera: Culicidae). Parasitol. Int..

[47] Bedini S., Flamini G., Cosci F., Ascrizzi R., Benelli G., Conti B. (2016). *Cannabis sativa* and *Humulus lupulus* Essential Oils as Novel Control Tools against the Invasive Mosquito *Aedes albopictus* and Fresh Water Snail *Physella acuta*. Ind. Crops Prod..

[48] Wanas A.S., Radwan M.M., Chandra S., Lata H., Mehmedic Z., Ali A., Baser K., Demirci B., ElSohly M.A. (2020). Chemical Composition of Volatile Oils of Fresh and Air-Dried Buds of *Cannabis chemovars*, Their Insecticidal and Repellent Activities. Nat. Prod. Commun..

[49] Pacheco-Hernández Y., Jonnathan Castro-Juárez C., Alberto Ramírez-García S., Cruz-Durán R., Lozoya-Gloria E., Villa-Ruano N. (2021). Volatiles from *Marina neglecta*: Biocide Effect on Insect Vectors of Tropical Diseases in Southern Mexico. J. Asia-Pac. Entomol..

[50] Sarma R., Adhikari K., Mahanta S., Khanikor B. (2019). Combinations of Plant Essential Oil Based Terpene Compounds as Larvicidal and Adulticidal Agent against *Aedes aegypti* (Diptera: Culicidae). Sci. Rep..

[51] Dambolena J.S., Zunino M.P., Herrera J.M., Pizzolitto R.P., Areco V.A., Zygadlo J.A. (2016). Terpenes: Natural Products for Controlling Insects of Importance to Human Health—A Structure-Activity Relationship Study. Psyche J. Entomol..

[52] Mantzoukas S., Ntoukas A., Lagogiannis I., Kalyvas N., Eliopoulos P., Poulas K. (2020). Larvicidal Action of Cannabidiol Oil and Neem Oil against Three Stored Product Insect Pests: Effect on Survival Time and in Progeny. Biology.

[53] McPartland J., Di Marzo V., De Petrocellis L., Mercer A., Glass M. (2001). Cannabinoid Receptors Are Absent in Insects. J. Comp. Neurol..

[54] Breer H., Hanke W., Benke D., Tareilus E., Krieger J., Maelicke A. (1989). Nicotinic Acetylcholine Receptors in the Nervous System of Insects. Molecular Biology of Neuroreceptors and Ion Channels.

[55] Stack G.M., Snyder S.I., Toth J.A., Quade M.A., Crawford J.L., McKay J.K., Jackowetz J.N., Wang P., Philippe G., Hansen J.L. (2023). Cannabinoids Function in Defense against Chewing Herbivores in *Cannabis sativa* L.. Hortic. Res..

[56] Martínez Rodríguez E.J., Phelan P.L., Canas L., Acosta N., Rakotondraibe H.L., Piermarini P.M. (2024). Larvicidal Activity of Hemp Extracts and Cannabidiol against the Yellow Fever Mosquito Aedes Aegypti. Insects.

[57] Park S.-H., Staples S.K., Gostin E.L., Smith J.P., Vigil J.J., Seifried D., Kinney C., Pauli C.S., Heuvel B.D.V. (2019). Contrasting Roles of Cannabidiol as an Insecticide and Rescuing Agent for Ethanol–Induced Death in the Tobacco Hornworm *Manduca sexta*. Sci. Rep..

