# Supplementary material for: Repellent, Lethal Activity, and Synergism of Cannabis sativa Extracts with Terpenes Against a Laboratory Colony of Triatoma infestans
Source: Plants (Basel). 2025 Oct 24;14(21):3258. doi: 10.3390/plants14213258 (PMC12610462; doi:10.3390/plants14213258)
Supplement: Supplementary file 1 [file plants-14-03258-s001.zip › plants-3885512-supplementary.pdf]

Laboratorio Hemp Lab
Juan B. Justo 4420, Mar del Plata, 7600
Tel: 1125713534

HEMP LAB

REPORT

CUSTOMER INFORMATION

Name/Company name	PLANTAR CIENCIA
e-mail	-
Celullar	2215733188
Type of analysis	CANNABINOIDS

INFORMACIÓN DE LA MUESTRA

Sample type	EXTRACT
ID muestra	EX02
Descripción de la muestra	Acetonic extract of Deep Mandarine
Sample arrival	04/04/22
Date of analysis	07/04/22

ANALYTICAL MÉTHODS

EQUIPMENT
High Performance Liquid Chromatography(HPLC)
Brand: Shimadzu
Model: LC-2050
MATERIALES
Reference standards: neutral mix with 8 components (CBC, CBD, CBDV, CBG, CBN, delta8THC, delta9THC, THCV)
Brand: Cerilliant

RESULTS

Cannabinoid	Concentration (mg/mL)	Concentration (%)
CBDV	**<1**	**<0.1**
CBG	**<1**	**<0.1**
CBD	**3.81**	**0.38**
THCV	**ND**	**ND**

CBN	**<1**	**<0.1**
delta9THC	**5.35**	**0.54**
Delta8THC	**ND**	**ND**
CBC	**<1**	**<0.1**

The concentration values, expressed in milligrams per milliliter (mg/mL), have to do with the amount of each of the cannabinoids present in the sample..

The values reported as concentration (%) have to do with the percentage amount of each cannabinoid present in the sample.

ND: No Detectable.

CHROMATOGRAM

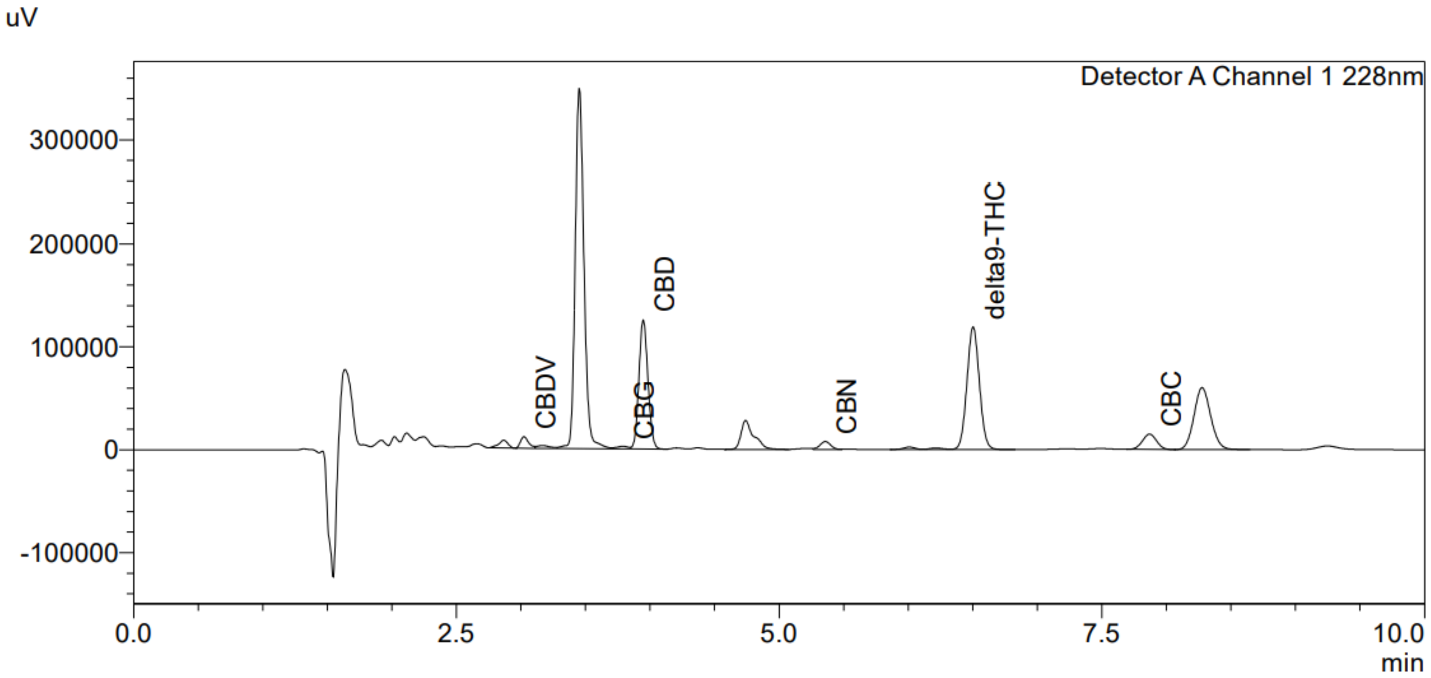

OBSERVATIONS:
THROUGH THE ANALYSIS OF THE CHROMATOGRAM, IT WAS POSSIBLE TO VISUALIZE THAT THE ACIDIC FORMS OF THE CANNABINOIDS CBD AND DELTA9-THC ARE PRESENT IN THE SAMPLE: CBDA AND THCA RESPECTIVELY.

The results contained in this document only apply to the samples tested as received in the laboratory.
The laboratory is not responsible for the information provided by the client
Partial reproduction of the report is prohibited without the written approval of the laboratory

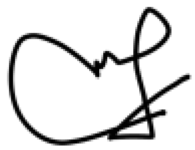

Pilar Díaz

Laboratorio Hemp Lab
Juan B. Justo 4420, Mar del Plata, 7600
Tel: 1125713534

REPORT

CUSTOMER INFORMATION

Nmae/Company name	PLANTAR CIENCIA
e-mail	-
Celullar	2215733188
Type of analysis	CANNABINOIDS

SAMPLE INFORMATION

Sample type	EXTRACT
ID sample	EX 03
Descriptión	Ethanolic extract of Deep Mandarine
Sample arrival date	04/04/22
Date of analysis	07/04/22

MÉTODO ANALÍTICO

EQUIPO
EQUIPMENT
High Performance Liquid Chromatography(HPLC)
Brand: Shimadzu
Model: LC-2050
MATERIALES
Reference standards: neutral mix with 8 components (CBC, CBD, CBDV, CBG, CBN, delta8THC, delta9THC, THCV)
Brand: Cerilliant

RESULTS

Cannabinoid	Concentration (mg/mL)	Concentration (%)
CBDV	**< LOQ**	**< LOQ**
CBG	**< LOD**	**< LOD**
CBD	**1.792**	**0.179**
THCV	**< LOD**	**< LOD**

CBN	**< LOQ**	**< LOQ**
delta9THC	**1.956**	**0.196**
Delta8THC	**< LOD**	**< LOD**
CBC	**0.621**	**0.062**

The concentration values, expressed in milligrams per milliliter (mg/mL), have to do with the amount of each of the cannabinoids present in the sample.The values reported as concentration (%) have to do with the percentage amount of each cannabinoid present in the sample. ND: No Detectable.

CROMATOGRAMA

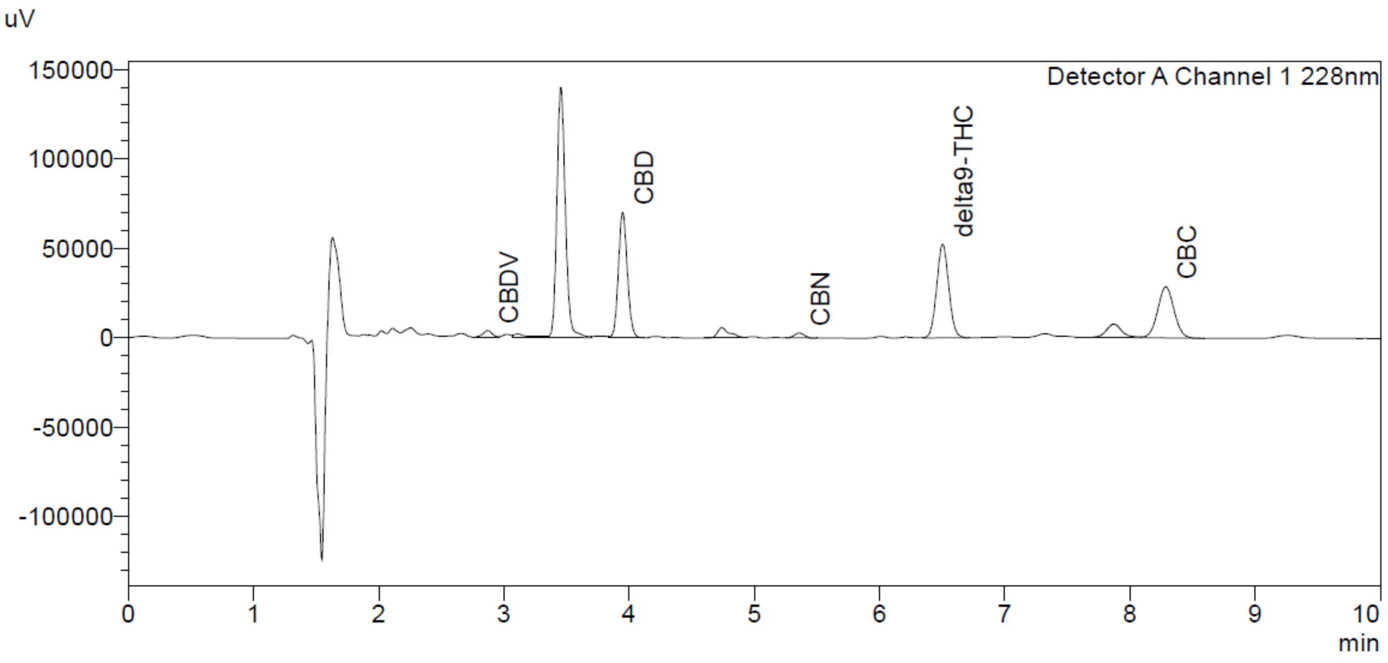

The results contained in this document only apply to the samples tested as received in the laboratory.
The laboratory is not responsible for the information provided by the client
Partial reproduction of the report is prohibited without the written approval of the laboratory

Pilar Díaz
Directora Técnica

Laboratorio Hemp Lab
Juan B. Justo 4420, Mar del Plata, 7600
Tel: 1125713534

HEMP LAB

REPORT

CUSTOMER INFORMATION

Name/Company name	PLANTAR CIENCIA
e-mail	plantarcienciaargentina@gmail.com
Cellular	-
Type of analysis	TERPENES

SAMPLE INFORMATION

Sample type	EXTRACTO CANNABIS
ID sample	EX 02
Sample Description	ACETONE EXTRACT OF DEEP MANDARINE
Sample arrival date	04/04/22
Date of Analysis	05/05/22

ANALYTICAL METHOD

EQUIPMENT
GC + detector FID
Brand: Shimadzu
Model: QP2020 NX
MATERIALS
Certified reference standards: mix of 19 terpenes (alpha-Pinene, Camphene, (-)-beta-Pinene, beta-Myrcene, delta-3-Carene, alpha-Terpinene, p-Cymene, d-Limonene, Ocimene, gamma-Terpinene, Terpinolene, Linalool, (-)-Isopulegol, Geraniol, beta-Caryophyllene, alpha-Humulene, Nerolidol, (-)-Guadiol, (-)-alpha-Bisabolol)
Brand: Restek

VALIDO PARA [illegible]TRA [illegible]LADA

RESULTADOS

Terpeno	Concentración (ppm)
Alpha-Pinene	**36.584**
Camphene	**ND**
(-)-Beta-Pinene	**149.792**
Beta-Myrcene	**ND**
Delta-3-Carene	**ND**
Alpha-Terpinene	**ND**
Ocimene 1	**ND**
d-Limonene	**178.431**
p-Cymene	**ND**
Ocimene 2	**278.486**
Gamma-Terpinene	**ND**
Terpinolene	**ND**
Linalool	**60.285**
(-)-Isopulegol	**ND**
Geraniol	**ND**
Beta-Caryophyllene	**418.159**
Alpha-Humulene	**140.994**
Nerolidol 1	**<25**
Nerolidol 2	**64.596**
(-)-Guaiol	**<25**
(-)-Alpha-Bisabolol	**95.529**

The concentration values, expressed in ppm, have to do with the amount of each of the terpenes that are present in the sample.

LOD (Limit of Detection): The limit of detection is the lowest concentration of analyte that can be detected, but not necessarily quantified, in a sample under established experimental conditions.

LOQ (Limit of Quantification): The limit of quantification is the lowest concentration of analyte that can be determined with precision and accuracy in a sample, under the established experimental conditions.

The results contained in this document only apply to the samples tested as received in the laboratory.
The laboratory is not responsible for the information provided by the client
Partial reproduction of the report is prohibited without the written approval of the laboratory

Pilar Díaz
Ing. Química

Laboratorio Hemp Lab
Juan B. Justo 4420, Mar del Plata, 7600
Tel: 1125713534

REPORT

CUSTOMER INFORMATION

Name/Company name	PLANTAR CIENCIA
e-mail	plantarcienciaargentina@gmail.com
Cellular	-
Type of analysis	TERPENES

SAMPLE INFORMATION

Sample type	CANNABIS EXTRACT
ID sample	EX 03
Description of the sample	ETANOLIC EXTRACT of DEEP MANDARINE
Date of arrival	04/04/22
Date of Analysis	05/05/22

ANALYTICAL METHOD

EQUIPMENT
GC + detector FID
Brand: Shimadzu
Model: QP2020 NX
MATERIALS
Certifed standards: mix of 19 terpenes (alpha-Pinene, Camphene, (-)-beta-Pinene, beta-Myrcene, delta-3-Carene, alpha-Terpinene, p-Cymene, d-Limonene, Ocimene, gamma-Terpinene, Terpinolene, Linalool, (-)-Isopulegol, Geraniol, beta-Caryophyllene, alpha-Humulene, Nerolidol, (-)-Guadiol, (-)-alpha-Bisabolol)
Brand: Restek

VALIDO PARA MUESTRA DETALLADA

RESULTADOS

Terpeno	Concentración (ppm)
Alpha-Pinene	**<25**
Camphene	**ND**
(-)-Beta-Pinene	**105.606**
Beta-Myrcene	**ND**
Delta-3-Carene	**ND**
Alpha-Terpinene	**ND**
Ocimene 1	**ND**
d-Limonene	**55.074**
p-Cymene	**ND**
Ocimene 2	**51.153**
Gamma-Terpinene	**ND**
Terpinolene	**ND**
Linalool	**<25**
(-)-Isopulegol	**ND**
Geraniol	**ND**
Beta-Caryophyllene	**138.995**
Alpha-Humulene	**50.137**
Nerolidol 1	**ND**
Nerolidol 2	**<25**
(-)-Guaiol	**<25**
(-)-Alpha-Bisabolol	**<25**

VÁLIDO PAR [illegible] DETALLADA

The concentration values, expressed in ppm, have to do with the amount of each of the terpenes that are present in the sample.
LOD (Limit of Detection): The limit of detection is the lowest concentration of analyte that can be detected, but not necessarily quantified, in a sample under established experimental conditions.
LOQ (Limit of Quantification): The limit of quantification is the lowest concentration of analyte that can be determined with precision and accuracy in a sample, under the established experimental conditions.

The results contained in this document only apply to the samples tested as received in the laboratory.
The laboratory is not responsible for the information provided by the client
Partial reproduction of the report is prohibited without the written approval of the laboratory

Pilar Díaz
Ing. Química
